# Inhibition of FAK Signaling Elicits Lamin A/C-Associated Nuclear Deformity and Cellular Senescence

**DOI:** 10.3389/fonc.2019.00022

**Published:** 2019-01-30

**Authors:** Hsiang-Hao Chuang, Pei-Hui Wang, Sheng-Wen Niu, Yen-Yi Zhen, Ming-Shyan Huang, Michael Hsiao, Chih-Jen Yang

**Affiliations:** ^1^Division of Pulmonary and Critical Care Medicine, Department of Internal Medicine, Kaohsiung Medical University Hospital, Kaohsiung Medical University, Kaohsiung, Taiwan; ^2^Division of Nephrology, Department of Internal Medicine, Kaohsiung Medical University Hospital, Kaohsiung Medical University, Kaohsiung, Taiwan; ^3^Department of Internal Medicine, E-Da Cancer Hospital, School of Medicine, I-Shou University, Kaohsiung, Taiwan; ^4^Genomics Research Center, Academia Sinica, Taipei, Taiwan; ^5^Department of Biochemistry, College of Medicine, Kaohsiung Medical University, Kaohsiung, Taiwan; ^6^Department of Internal Medicine, Kaohsiung Municipal Ta-Tung Hospital, Kaohsiung Medical University, Kaohsiung, Taiwan; ^7^Department of Respiratory Therapy, College of Medicine, Kaohsiung Medical University, Kaohsiung, Taiwan

**Keywords:** non-small cell lung cancer, senescence, focal adhesion kinase, nuclear deformity, lamin A/C

## Abstract

Focal adhesion kinase (FAK) is a non-receptor kinase that facilitates tumor aggressiveness. The effects of FAK inhibition include arresting proliferation, limiting metastasis, and inhibiting angiogenesis. PF-573228 is an ATP-competitive inhibitor of FAK. Treating lung cancer cells with PF-573228 resulted in FAK inactivation and changes in the expressions of lamin A/C and nuclear deformity. Since lamin A/C downregulation or deficiency was associated with cellular senescence, the senescence-associated β-galactosidase (SA-β-gal) assay was used to investigate whether PF-573228 treatment drove cellular senescence, which showed more SA-β-gal-positive cells in culture. p53 is known to play a pivotal role in mediating the progression of cellular senescence, and the PF-573228-treated lung cancer cells resulted in a higher p53 expression level. Subsequently, the FAK depletion in lung cancer cells was employed to confirm the role of FAK inhibition on cellular senescence. FAK depletion and pharmacological inhibition of lung cancer cells elicited similar patterns of cellular senescence, lamin A/C downregulation, and p53 upregulation, implying that FAK signaling is associated with the expression of p53 and the maintenance of lamin A/C levels to shape regular nuclear morphology and manage anti-senescence. Conversely, FAK inactivation led to p53 upregulation, disorganization of the nuclear matrix, and consequently cellular senescence. Our data suggest a new FAK signaling pathway, in that abolishing FAK signaling can activate the senescence program in cells. Triggering cellular senescence could be a new therapeutic approach to limit tumor growth.

## Introduction

Focal adhesion kinase (FAK) is a multifunctional non-receptor tyrosine kinase that participates in a variety of signaling axes (1–5). In response to extracellular stimuli, FAK translocates to the focal adhesion complex, and mediates molecular signaling for cellular events (6–9). In focal adhesion, FAK cascades signal the focal adhesion complex to promote cell proliferation and migration (4, 9, 10). In addition to the focal adhesion complex and cytoplasm, FAK is also present in the cell nucleus (3, 11). Nuclear FAK acts as a cotranscriptional factor in gene transcription and is involved in p53 degradation, in contrast to its enzymatic function in protein phosphorylation (3, 11, 12). Whereas, FAK in the focal adhesion complex affects the expressions of cyclin B1 and cyclin D1 to program tumor cell proliferation (6, 13, 14), nuclear FAK elicits p53 degradation to drive cell cycle progression (11, 12).

The biological roles of FAK in cell migration and proliferation have also been implicated in pathological progression and in the development of cancer. There are several lines of evidence suggesting that FAK activity can manipulate tumor phenotypes, leading to uncontrolled proliferation, neovascularization, and metastasis (5, 15, 16), and the FAK signaling to tumor cell propagation represents tumorigenic capacity (5, 11). Given the crucial roles of FAK in these malignant processes, FAK is regarded to be a potential target for anti-cancer therapy (17, 18). Experiments have shown that FAK depletion results in silencing of cancer-promoting gene expressions in human hepatocellular carcinoma (HCC) xenotransplants in nude and severe combined immunodeficiency (SCID) mice (19). Moreover, the enzymatic function of FAK involves in proliferation and metastasis (9, 15, 20). Suppressing the catalytic activity of FAK or sequestering FAK in the cytoplasm has been reported to potentially perturb FAK signaling, which implies that chemical inhibitors of the enzymatic activity of FAK may be a pharmacological strategy to limit cancer growth and metastasis (6, 7, 21, 22).

Triggering cell apoptosis and arresting cell cycle progression with pharmacological regimens are common strategies to limit tumor cell growth. FAK inhibition represents an anti-cancer therapeutic strategy, as FAK inhibitors have effects on anti-angiogenesis, anti-proliferation, and anti-invasion effects (5, 15, 23). Besides, inducing cellular senescence in tumor cells could be a new therapeutic approach to limit tumor cell growth (24). Although therapy-induced senescence (TIS) in cancer cells may result from deficient apoptosis (25), driving cancer cells to cellular senescence could be a way to limit tumor propagation (26). In general, chemotherapy-induced DNA damage, telomere shortening, and oncogenic stress are the three main pathological causes of senescence (24, 27–32), and the induction of cellular senescence with these drug regimens is a side effect. Cases of β-gal-positive lung cancer biopsies in response to chemotherapy have been reported (24, 29, 33). Inducing cellular senescence could be a new approach to limit cancer growth based on phenotypic aging without DNA damage or genomic instability (31, 34). Chromatin or nuclear skeleton disorganization could be a cause of cellular senescence instead of oncogenic stress and replicative failure (8, 26).

Recent pharmacological advances in cancer therapy have led to an increased focus on developing chemical compounds that are able to target specific molecules in tumor cells to both improve efficacy and lower toxicity (4, 35). PF-573228, which competes with ATP binding to abolish the catalytic function of FAK, can inhibit the phosphorylation of FAK at tyrosine 576/577 and FAK kinase function (36, 37). Consequently, PF-573228 efficiently suppresses both the growth and metastasis of epithelial carcinoma (4, 36). The pharmacological effects of PF-573228 have been characterized based on the inhibition of FAK catalytic activity (36). In this study, we hypothesized that inhibiting the enzymatic function of FAK would stop lung cancer cell growth and invasion. Interestingly, the enzymatic inactivation of FAK resulted in nuclear deformity. When we investigated the cause and effect of nuclear deformity by PF-573228, we observed that p53 upregulation, lamin A/C downregulation, and cellular senescence in the lung cancer cells exposed to PF-573228. Strikingly, perturbation of FAK signaling led to downregulation of lamin A/C and cellular senescence rather than proliferative arrest, and halted migration of the lung cancer cells. These results showed that treatment with a FAK inhibitor could be a therapeutic approach to abrogate tumor growth. In addition, these findings revealed the crucial role of FAK signaling in anti-senescence, and that inhibition of FAK resulted in the progression of senescence.

## Materials and Methods

### Materials

Detailed information on the materials is listed in Supplementary Table S1.

### Cell Culture and Drug Treatment

A549 cells, H1299 cells, and H460 cells were purchased from ATCC. The cells were cultured in RPMI 1640 medium supplemented with 10% fetal bovine serum (FBS) at 37°C in a humidified atmosphere at 5% CO_2_, and treated with PF-573228 (TOCRIS, Bristol, UK) at concentrations of 0, 0.1, 1, or 10 μM.

### Western Blot Analysis

The cells were harvested and lysed in 1x RIPA buffer (Merck, Darmstadt, Germany) containing protease and phosphatase inhibitors. The protein concentration was determined using a Bio-Rad DC protein assay kit (Bio-Rad, California, USA). For Western blot analysis, 30 μg of total protein was applied to SDS-PAGE and transferred to a PVDF membrane. The membranes were blocked in 5% skim milk for 2 h in TBST buffer (20 mM Tris-Cl, 150 mM NaCl, 0.1% Tween 20, pH 7.4). After blocking, the membranes were probed with the primary antibody overnight. Antibodies against FAK, p-FAK, cyclin B1, p53, and lamin A/C were used in immunoblotting. The given protein bands were identified by horseradish peroxidase-conjugated secondary antibodies and developed with an enhanced chemiluminescence solution.

### Flow Cytometry Cell Cycle Analysis

The cells were harvested and washed with PBS buffer, and then fixed in 70% (v/v) ethanol. The fixed cells were stained with propidium iodide solution and injected into an Attune NxT Flow Cytometer (Life Tech, California, USA) to analyze the cell cycle profile.

### Immunofluorescent Staining and Immunofluorescent Microscopic Imaging

A Leica DMi8S epifluorescence microscope (Wetzlars, Germany) equipped with an X-Cite XCT10A (Lumen Dynamics, Wiesbaden, Germany) light source, filters and objectives (10x, 20x, 40x, and 63x) was used to observe fluorescent signals in the cells. In addition to epifluorescence, confocal images were captured using an OLYMPUS FV1000 confocal laser scanning microscope equipped with a light source, filters and objectives (10x, 20x, 40x, 63x, and 100x). Cells were seeded on 12-mm coverslips in a 24-well culture plate. The cells were harvested and fixed in 4% paraformaldehyde in PBS for 10 min, and permeabilized in 0.5% Triton in PBS for 5 min. After fixation, the cells were subjected to immunofluorescent staining with antibodies recognizing FAK and emerin. Phalloidin-TRITC was used as an additional reagent. Cell nuclei were stained with 0.2 μg/mL 4′, 6-diamidino-2-phenylindole (DAPI).

### Senescence-Associated β-Galactosidase Staining

The cells were fixed with 4% paraformaldehyde for 15 min. After fixation, acidic β-galactosidase (SA-β-gal) was assayed in senescence assay buffer (1 mg/mL 5-bromo-4-chloro-3-indolyl β D-galactopyranoside (X-gal), 5 mM K_3_Fe(CN)_6_, 5 mM K_4_Fe(CN)_6_, 2 mM MgCl_2_, 150 mM NaCl, 40 mM citric acid, and 40 mM Na_2_HPO_4_ at pH 6.0) in the dark at 37°C for 16 h. SA-β-gal activity was detected based on SA-β-gal-hydrolyzed X-gal, which produces a blue color. All chemical reagents were purchased from Sigma-Aldrich.

### Cell Growth Assay

The cells were trypsinized, resuspended in 1xPBS, and stained with trypan blue (Sigma-Aldrich). The number of cells was counted with a hemocytometer.

### Lentiviral Production and Infection

Lentivirus-associated plasmids encoding luciferase, and FAK short hairpin RNA (shRNA) were obtained from the National RNAi Core Facility of Academia Sinica, Taiwan. The production and infection of lentiviruses were performed according to the guidelines of the National RNAi Core Facility.

### Statistical Analysis

The experimental data were digitized and analyzed. Data are presented as the mean ± the standard error of the mean (SEM). One-way ANOVA was used to compare digitized data and measurements from independent experiments in two or more groups, and the Student's *T*-test was used to compare two independent samples. A *p* < 0.05 was considered to indicate a statistically significant difference.

## Results

### PF-573228 Causes Cessation of the Propagation of Lung Cancer Cells

**Figure 1:**
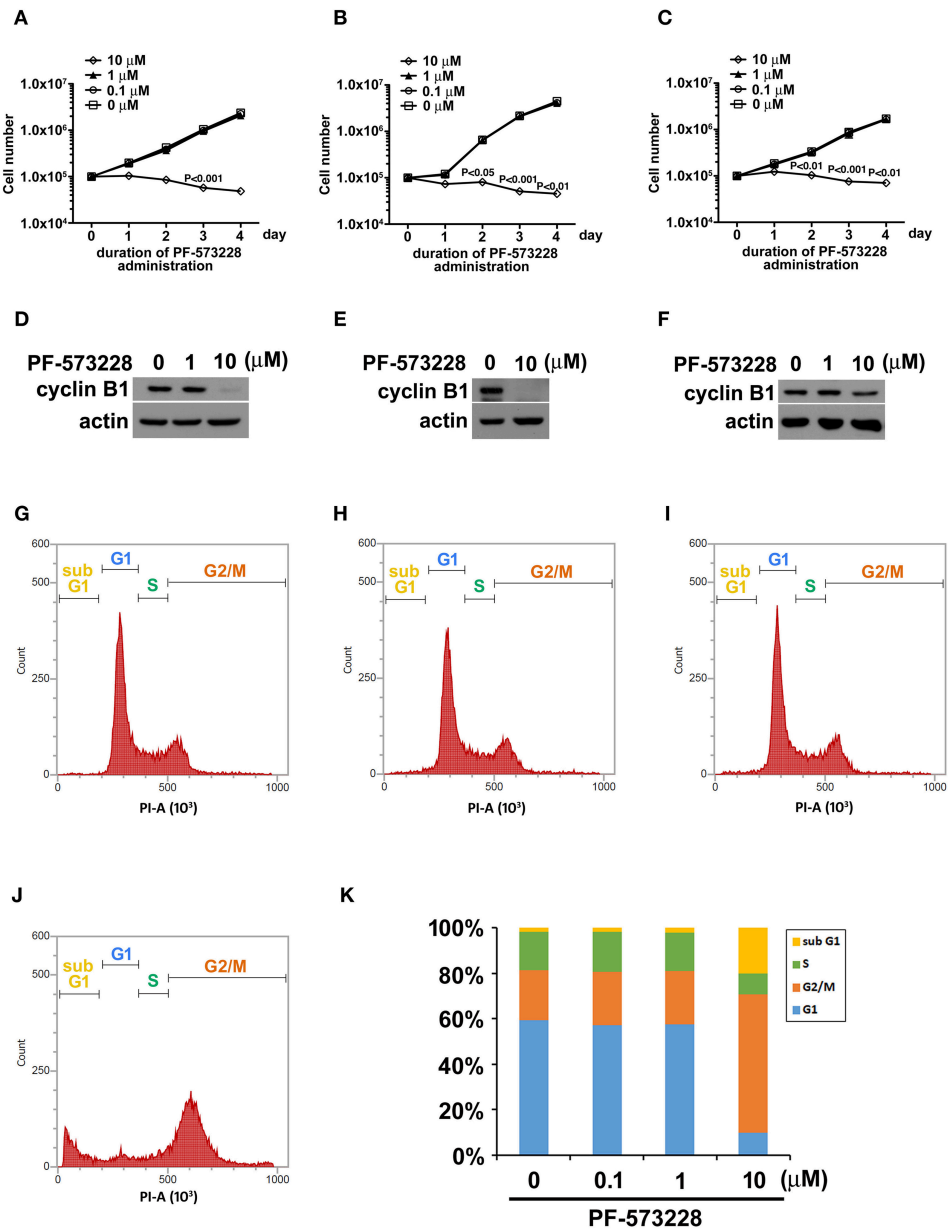
PF-573228 inhibited lung cancer cell growth. Three different types of lung cancer cells, **(A)** A549 lung adenocarcinoma and **(B)** H460, and **(C)** H1299 large cell carcinoma, were selected for the PF-573228 administration regimen. Cell growth curves of the three lung cancer cell lines treated with various doses of PF-573228 for 4 days were established. The administration of PF-573228 at 10 μM to the lung cancer cells effectively suppressed cell growth *in vitro*, as proliferative activity totally ceased in the cells exposed to 10 μM PF-573228. **(D)** On the third day, PF-573228-treated cells were harvested and subjected to Western blot analysis for cyclin B1. Cyclin B1 levels were much higher in A549 cells with 1 μM PF-573228 or without PF-573228 treatment than in the cells treated with higher concentrations of PF-573228. **(E)** After 10 μM PF-573228 treatment, cyclin B1 levels declined markedly in H460 cells. **(F)** PF-573228 administration slightly reduced cyclin B1 levels in H1299 cells. A549 cells were harvested and subjected to flow cytometry analysis for cell cycle profiling after PF-573228 treatment for 3 days. The concentrations of PF-573228 were 0 μM **(G)**, 0.1 μM **(H)**, 1 μM **(I)** and 10 μM **(J)**, respectively, **(K)** After 10 μM PF-573228 treatment, the G2/M ratio was significantly extended. The apoptotic ratio was also increased in A549 cells with 10 μM PF-573228 treatment.

Focal adhesion signaling is involved in cell proliferation, and FAK plays a key role in the focal adhesion complex that relays focal adhesion signals to the cell proliferation program (9, 15). Given the role of FAK signaling in tumor growth and metastasis, we hypothesized that inhibiting the catalytic activity of FAK may disrupt FAK signaling and blunt tumor cell proliferation. Therefore, we treated three distinct non-small cell lung cancer cell lines (A549 lung adenocarcinoma cells and H460 and H1299 large cell carcinoma cells) with PF-573228, an enzymatic inhibitor of FAK. PF-573228 was administered to the lung cancer cells for 4 days at three doses: 0.1, 1, or 10 μM. The growth curves showed that 10 μM PF-573228 effectively induced cessation of cell growth (Figures 1A–C).

We then examined the expression level of the cell cycle regulator cyclin B1, which has been reported to be a downstream effector of FAK signaling. Western blot analysis showed that cyclin B1 expression levels were much lower after the cells were exposed to 10 μM PF-573228 (Figures 1D–F). To further characterize the effect of PF-573228 treatment on cell cycle progression, we analyzed the cell cycle distribution using flow cytometry analysis. The results showed that a low PF-573228 concentration had little influence on cell cycle progression (Figures 1G–I), whereas a high PF-573228 concentration (10 μM) halted cell cycle progression at the G2/M transition (Figures 1J,K). This showed that PF-573228 treatment effectively suppressed multiplication of lung cancer cells.

### PF-573228 Administration Inactivates FAK

**Figure 2:**
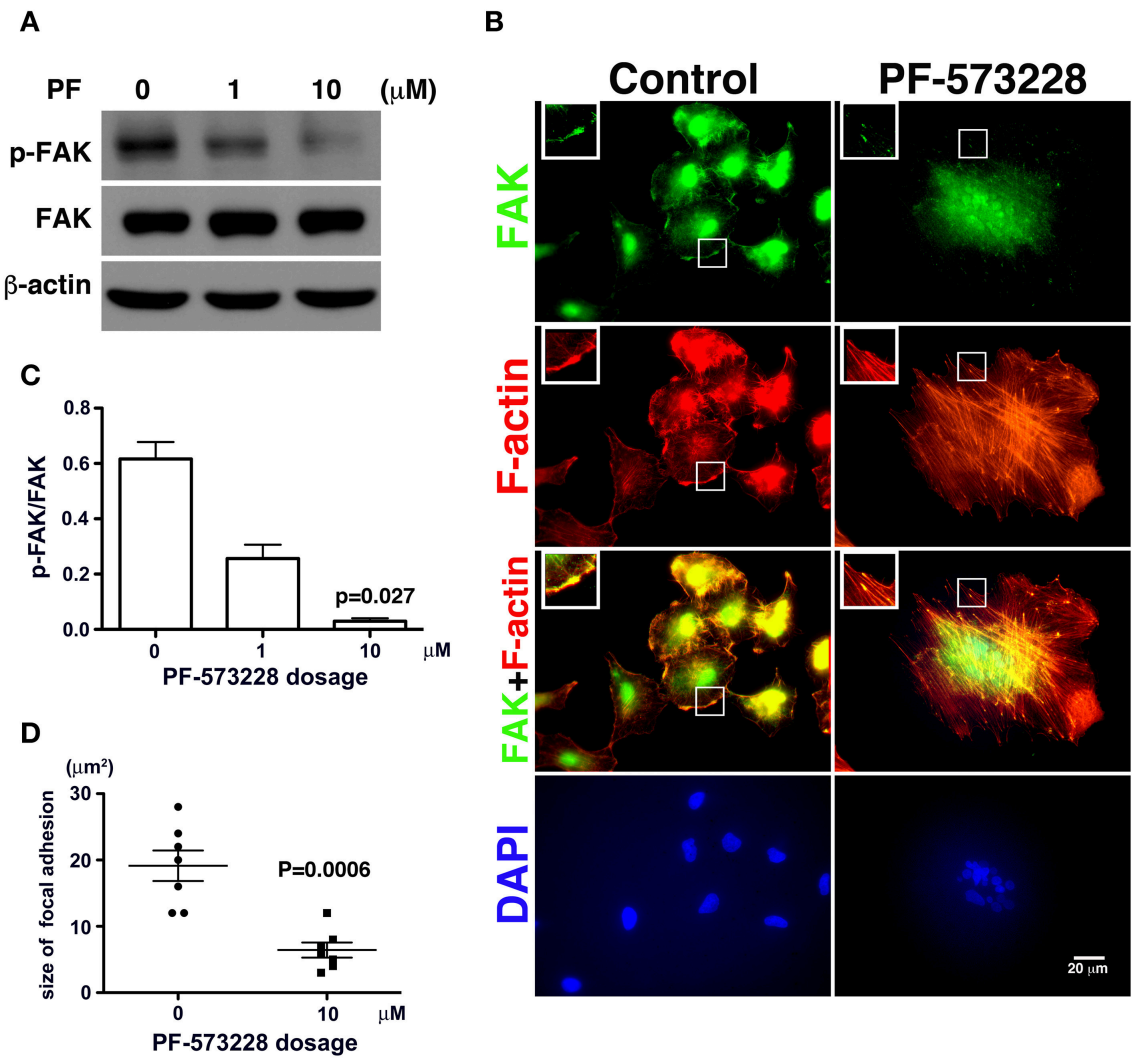
PF-573228 as a catalytic inhibitor inactivated the kinase function of FAK. **(A)** FAK expression levels and FAK activity, as measured by the phosphorylation of FAK at tyrosine 576 and 577, were quantified by Western blot analysis after treatment of lung cancer cells with PF-573228 for 3 days. **(B)** The cells were stained with phalloidin to visualize F-actin (red) and a FAK antibody to visualize the FAK distribution (green). In cells without PF-573228 administration, FAK translocated to focal adhesions at the tips of actin stress fibers, and the focal adhesions were relatively large. When cells were exposed to 10 μM PF-573228, FAK translocation to focal adhesions was reduced, and the sizes of the focal adhesions were smaller. Nuclei in cells treated with PF-573228 were deformed, as visualized with DAPI staining, whereas most nuclei in cells without PF-573228 treatment were oval shaped. **(C)** The p-FAK/FAK ratios in the cells with exposure to 1 μM and 10 μM PF-573228 were reduced to less than half and one tenth compared with the cells without PF-573228 treatment, respectively. **(D)** The sizes of FAK-based focal adhesions were 19 μm^2^ on average in the cells without PF-573228 treatment and 6.4 μm^2^ on average in the cells without PF-573228 treatment.

Since phosphorylation of FAK at Tyr-576 and Tyr-577 (p-FAK) represents enzymatic activation of FAK (37), an antibody against p-FAK was used to confirm the kinase activity of FAK and verify the effect of PF-573228 on FAK inactivation. FAK activity was practically blocked by 10 μM PF-573228 in A549 cells (Figures 2A,C). To further confirm the inactivation of FAK by PF-573228 treatment, we also examined the phosphorylation of tyrosine 397 in FAK (pTyr-397). The results showed that the intensity of pTyr-397 was decreased after PF-573228 treatment (Figure S1). FAK is a key regulator of integrin signaling for focal adhesion assembly (38, 39). In addition to FAK inhibition, PF-573228 has been shown to perturb integrin-based signaling for focal adhesion maturation (36).

Treatment of the lung cancer cells with PF-573228 resulted in failure of FAK activation, and translocation to focal adhesion was observed in immunofluorescent imaging (Figure 2B). When the cells were cultured in PF-573228-free medium, more FAK translocated to focal adhesions, which appeared as plaque-like patterns in the cell periphery that formed at the tips of stress fibers, as visualized in cells stained with phalloidin-labeled F-actin and an antibody against FAK (Figure 2B). By contrast, in the A549 cells treated with PF-573228, only a few FAK molecules translocated to focal adhesions, and tiny FAK-based focal adhesions formed at the tips of F-actin bundles, indicating failure of focal adhesion maturation (Figure 2B). The sizes of focal adhesions were measured and the areas of FAK at the tips of the actin stress fibers were digitized using Image Pro software. The sizes of focal adhesions ranged from 12 to 28 μm^2^ in the cells without PF-573228 treatment, whereas the extent of FAK-based focal adhesion was approximately 3–12 μm^2^ after PF-573228 treatment (Figure 2D).

### Aberrant Nuclear Appearance and Lamin A/C Downregulation Occur Concurrently in the Lung Cancer Cells Exposed to PF-573228

**Figure 3:**
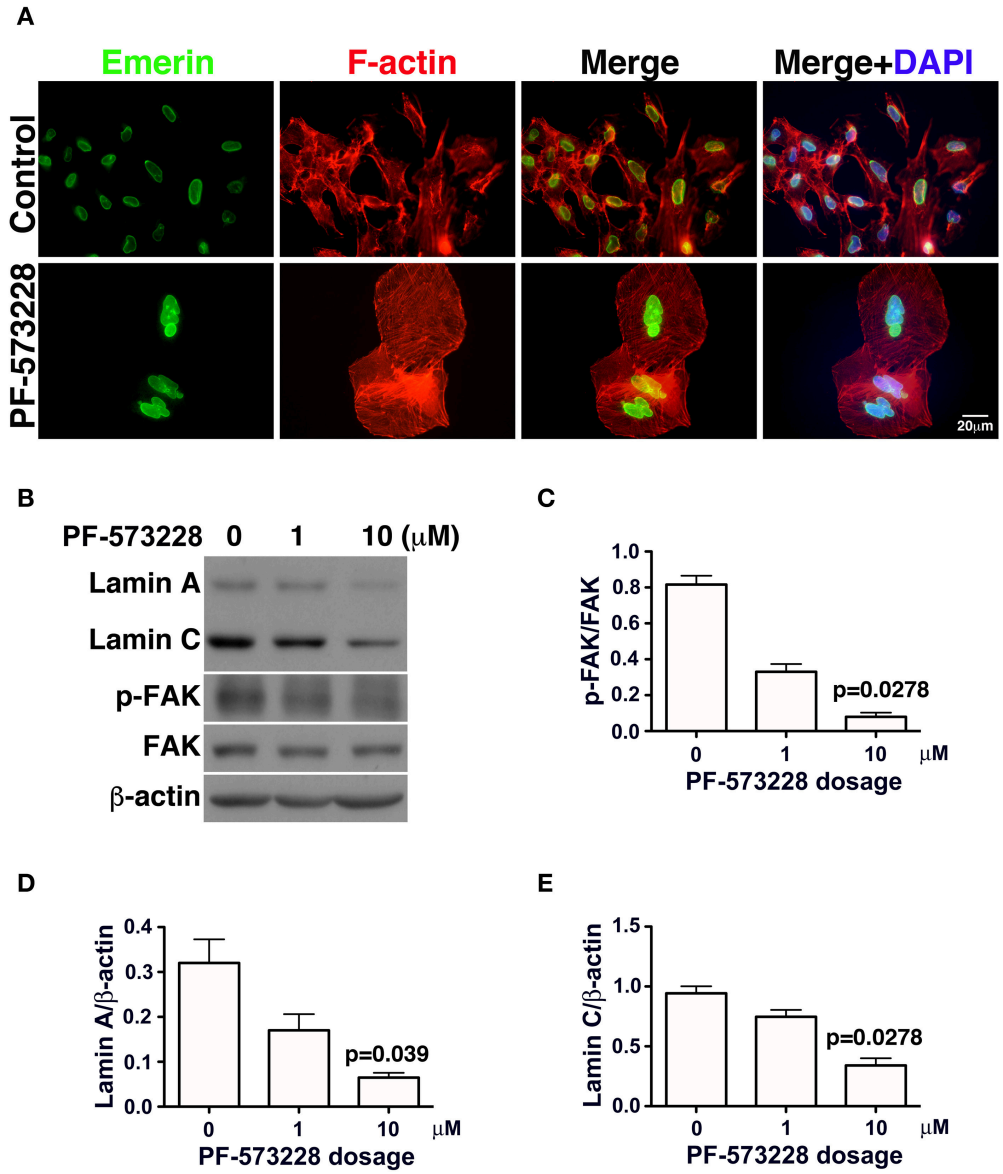
Downregulation of lamin A and lamin C and nuclear deformity in A549 cells exposed to PF-573228. **(A)** After PF-573228 treatment of A549 cells, the cells were fixed and stained with phalloidin to label F-actin (red) and an antibody against emerin (green) to outline the nuclear shape. Cells treated with PF-573228 were extremely large and had deformed nuclei, whereas mostly oval-like nuclei were present in the cells without PF-573228 treatment. **(B)** The cells treated with 10 μM PF-573228 exhibited a decrease in p-FAK levels. Lamin A and lamin C expression levels were much lower in A549 cells exposed to 10 μM PF-573228. **(C)** The p-FAK/FAK ratios in A549 cells exposed to 1 μM and 10 μM PF-573228 were less than half and one tenth of those in A549 cells without PF-573228 treatment, respectively. **(D,E)** Decreased lamin A and lamin C levels appeared in A549 cells treated with PF-573228.

In the absence of PF-573228, most cells contained oval or round nuclei, as visualized by DAPI staining (Figure 2B). Interestingly, a distorted nuclear morphology was observed in the A549 cells treated with PF-573228 (Figure 2B). As DAPI staining was insufficient to clearly visualize the nuclear appearance in detail, an antibody against emerin (40), a nuclear inner membrane protein, was used to visualize the nuclear shape in the PF-573228-treated A549 cells. Emerin staining revealed that the cells without PF-573228 treatment harbored oval-like nuclei. By contrast, most nuclei were larger and had irregular shapes with invagination in the cells upon exposure to 10 μM PF-573228 (Figure 3A).

Lamin A/C is the nuclear skeleton responsible for maintaining and stabilizing the nuclear architecture (41–44). Because interrupting FAK signaling resulted in nuclear deformity (Figure 2B), changes in lamin A/C expression levels were assessed. The effects of FAK signaling on the expressions of lamin A/C and other nuclear skeletal proteins inferred that the nuclear deformity caused by PF-573228 was attributable to changes in lamin A/C expression.

To investigate changes in lamin A/C expressions in cells exposed to PF-573228, PF-573228-treated cells were harvested and subjected to Western blot analysis. Inhibition of FAK activity led to lower p-FAK levels (Figures 3B,C) and deformed nuclei in the lung cancer cells (Figure 3A). To quantify the expressions of lamin A and C, the intensities of their protein bands were normalized to β-actin (Figure 3B). The expressions of lamin A and C were much lower in the A549, H460, and H1299 cells treated with PF-573228 compared to those without PF-573228 treatment (Figure 3B and Figures S2A,B). The lamin A and C band intensities were quantified and plotted in bar charts. The ratios of lamin A/β-actin and lamin C/β-actin in A549 cells exposed to PF-573228 were reduced by one third and one half, respectively, compared to A549 cells without PF-573228 treatment (Figures 3D,E). Similar trends of downregulation of lamin A and lamin C by PF-573228 treatment were also detected in the two other lung cancer cell lines (Figures S2A,B).

### Lung Cancer Cells Are Destined to Senescence After Inhibition of FAK Enzymatic Function

**Figure 4:**
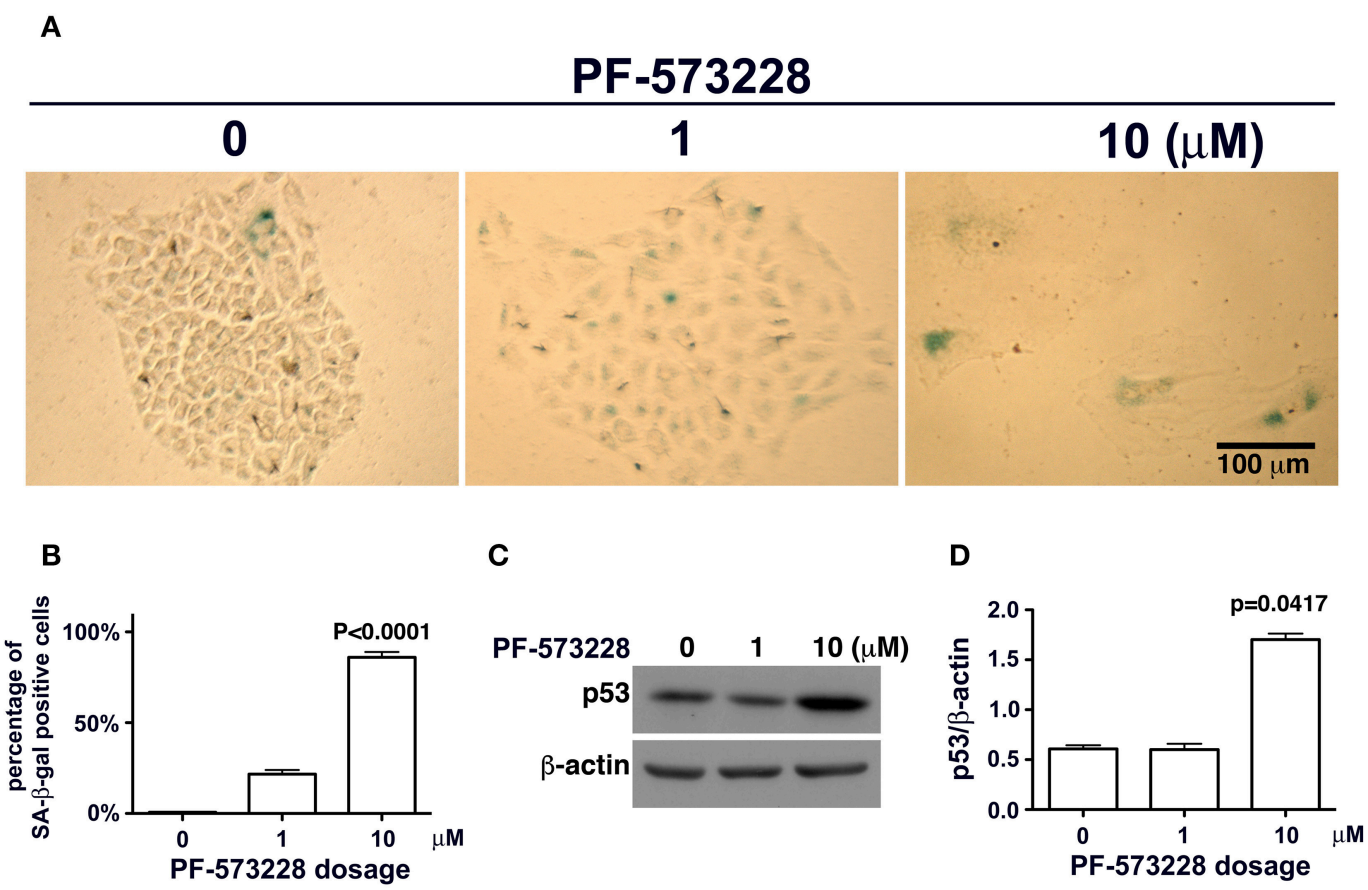
Cellular senescence occurred in lung cancer cells after FAK inhibition. **(A)** A549 cells were exposed to 0, 1 μM, or 10 μM PF-573228 for 7 days. SA-β-gal-positive cells appeared sporadically in cells without PF-573228 treatment. The cells treated with 1 μM PF-573228 were slightly enlarged, with few β-gal-positive cells. The cells treated with 10 μM PF-573228 were quite large, and most were β-gal positive. **(B)** The ratio of SA-β-gal-positive cells to the total population was calculated and plotted in a bar chart. SA-β-gal-positive cells represented < 1% of the total A549 cell population without PF-573228 treatment, ~21% in the 1 μM PF-573228-treated A549 cell population, and more than 80% in the 10 μM PF-573228-treated A549 cell population. **(C)** A549 cells were treated with 0, 1, or 10 μM PF-573228 for 4 days. p53 was not obviously increased in 1 μM PF-573228 treated-A549 cells and was significantly elevated in 10 μM PF-573228-treated A549 cells. **(D)** p53 levels approximately tripled in A549 cells exposed to 10 μM PF-573228 compared to cells with or without 1 μM PF-573228 treatment.

Mutant LMNA, mutations that affect lamin A/C expression, and lamin A/C depletion in cells have been associated with premature aging and cellular senescence (8, 30, 32, 42, 45). Based on the concurrent lamin A/C downregulation and nuclear deformity observed in lung cancer cells exposed to PF-573228 (Figures 3A,B), we examined the development of cellular senescence in lung cancer cells treated with PF-573228. The SA-β-gal activity in cells was assayed by *in situ* staining using the chromogenic substrate X-gal, which colored SA-β-gal-positive cells blue. As noted in Figure 4A, blue cells were clearly visible in the cells treated with PF-573228 (Figure 4A), whereas a sporadic distribution of blue-colored cells was observed in the cells without PF-573228 treatment (Figure 4A). The bar chart in Figure 4B shows that nearly 90% of the cells exposed to a higher dose of PF-573228 were positive for SA-β-gal, compared to ~20% of the cells exposed to a lower dose of PF-573228, and ~1% of the cells without PF-573228 treatment.

### Upregulation of p53 in Cells Exposed to PF-573228

Disruption of FAK signaling by PF-573228 caused cellular senescence. However, the mechanisms by which inhibition of FAK signaling affects senescence programming remain unclear. Cellular senescence in chemotherapy-affected cancer cells has been observed in several studies (24, 29, 46). In addition, clinical studies have reported that p53 plays a role in the development of cellular senescence in chemotherapy-affected cancer cells (46, 47). p53 is known to be a transcription factor in programed senescence and cell cycle arrest (48), and it may play a similar role in the cellular senescence program in lung cancer cells exposed to PF-573228 as in cells in which FAK signaling is interrupted.

To investigate whether or not p53 plays a role in PF-573228-induced cellular senescence, p53 expression levels were examined in PF-573228-treated cells. Western blot analysis showed that p53 expression levels increased significantly by more than 3-fold compared to cells without PF-573228 treatment and cells treated with a low concentration of PF-573228 (Figures 4C,D, and Figure S3).

### Engagement of FAK Signaling With Nuclear Integrity and p53 Expression

FAK is not the only molecule targeted by PF-573228 (36). Although FAK enzymatic activity was blocked by PF-573228 administration, off-target effects could also have turned off other kinases, for example, cyclin-dependent kinases. Therefore, signaling perturbations of other kinases may have caused the pathogenic senescence in the lung cancer cells.

**Figure 5:**
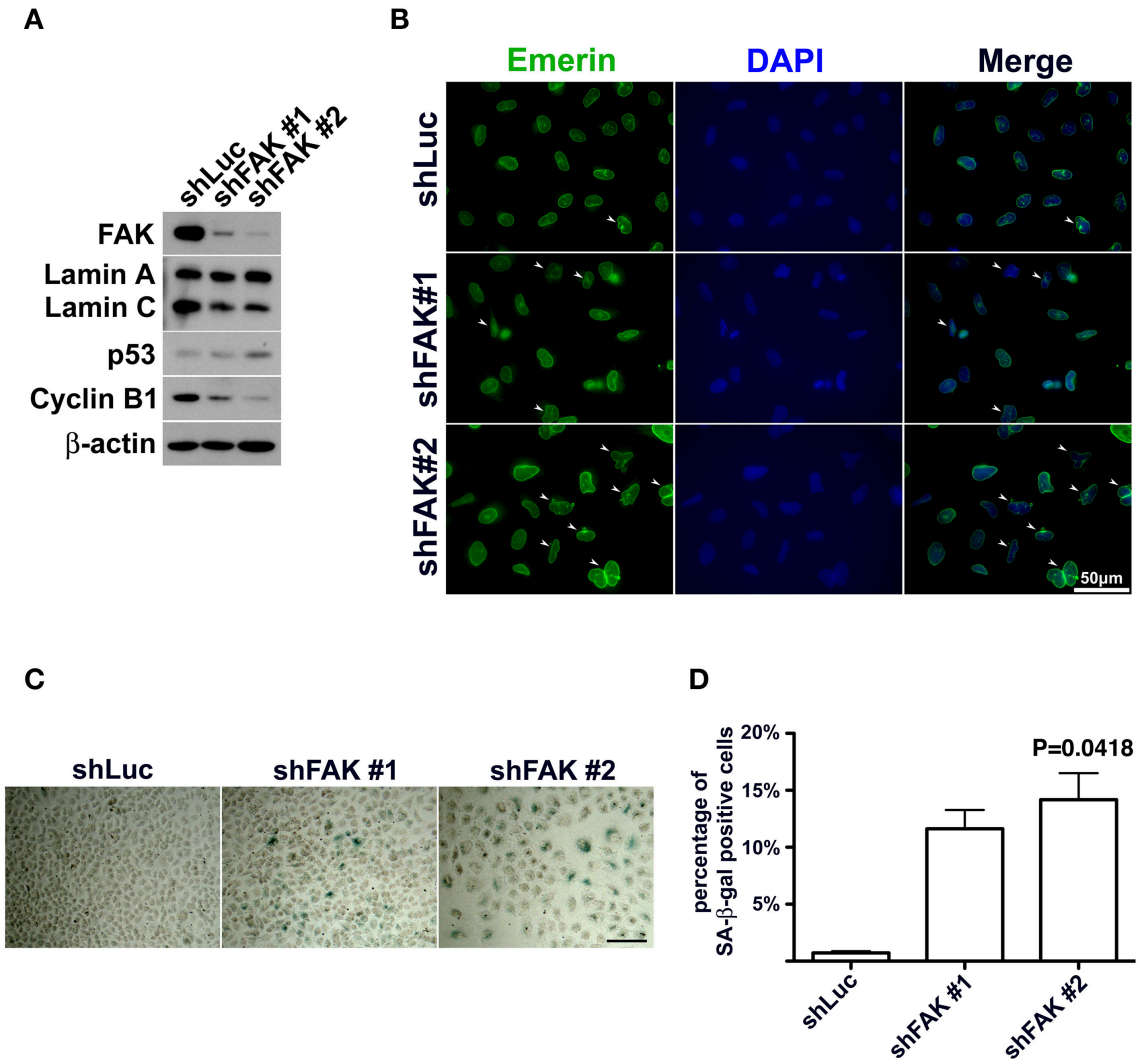
FAK depletion resulted in nuclear deformity and cellular senescence. **(A)** A549 cells with FAK depletion by shRNA were seeded and incubated for 7 days. Western blot analysis revealed low FAK levels in the cells with shFAK and higher levels in the cells with shLuc. Upon FAK depletion, lamin C and cyclin B1 levels decreased, and p53 expression levels increased. **(B)** The cells were fixed and stained with an antibody against emerin (green) to outline the nuclear shape. Cells with FAK depletion were slightly larger, with a higher proportion of deformed nuclei (arrowhead), whereas mostly oval-like nuclei were present in cells without FAK depletion. (Scale bar, 50 μm) **(C)** SA-β-gal-positive cells were sporadically visible in A549 cells with shLuc. By contrast, more SA-β-gal-positive cells were observed among cells with shFAK. (Scale bar, 100 μm) **(D)** The bar chart shows that < 1% of the cells in the shLuc population were SA-β-gal-positive, whereas more than 10% of the shFAK cell population was SA-β-gal positive.

If FAK has an anti-senescence effect, FAK depletion would cause anti-senescence to fail and escalate senescence programing. To clarify the role of loss of FAK signaling in the development of cellular senescence and nuclear deformity with changes in lamin A and lamin C expressions, we used an shRNA targeting FAK to deplete the expression of FAK in lung cancer cells. After introducing shFAK into lung cancer cells, cells harboring shFAK were selected. To assess FAK knockdown, two shFAK clones were selected for Western blot analysis and senescence assays, which showed that shFAK successfully caused FAK depletion in A549, H460, and H1299 cells (Figure 5A and Figure S4A). In addition, the impact of FAK depletion on the downregulation of lamin C and cyclin B1 and upregulation of p53 was validated (Figure 5A and Figure S4A). We also assessed nuclear appearance using emerin staining, which revealed that A549 cells without FAK depletion harbored oval-like nuclei. By contrast, the ratio of nuclei harboring slightly larger and irregular shapes was increased in the cells with FAK depletion (Figure 5B). Similar results were obtained in the H460 and H1299 cells (Figures S4B,C). In addition, lung cancer cells harboring shLuc or shFAK were subjected to senescence assays, which revealed more SA-β-gal-positive A549 cells harboring shFAK (Figure 5C). By contrast, few SA-β-gal-positive cells were visible in those harboring shLuc. The SA-β-gal-positive cells represented ~0.7% of the shLuc A549 population. In the two shFAK cell clones, SA-β-gal-positive cells represented ~11 and 15%, respectively (Figure 5D). Similar results were observed in H460 and H1299 cells (Figures S4D–F). However, H460 cells grew in single and multiple layers (Figure S4D), and it was difficult to measure the ratio of SA-β-gal-positive cells.

### Senescent Cells Reactivate Their Proliferative Activity After PF-573228 Withdrawal From Cell Culture

Aging and cellular senescence are often present in replicative failure, oncogenic induction, and telomere shortening (8, 34, 49). In clinical cases, chemotherapy or radiation has been shown to induce cellular senescence, and DNA damage or genomic instability is thought to be the pathological cause. Therapy-induced senescence can be classified as replicative senescence (24, 27, 33, 48), and replicative senescence is able to cease tumor growth (47, 50). However, this cellular senescence is reversible (31, 51, 52). Senescent cells expressing low levels of p16 have been shown to reversibly exit senescence when p53 expression levels fall (52).

**Figure 6:**
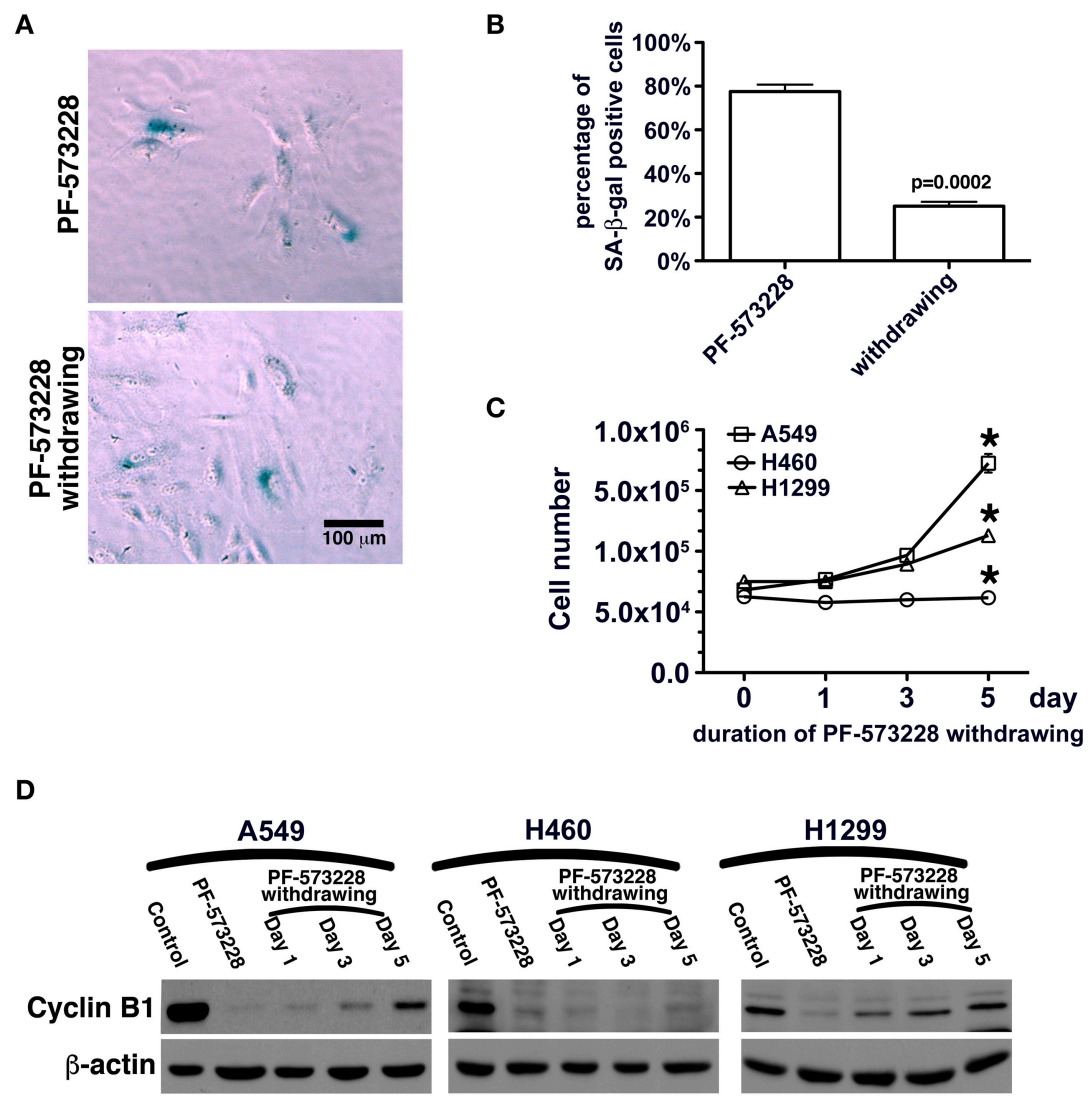
Recovery of the proliferative activity of lung cancer cells after PF-573228 withdrawal. **(A)** The population of SA-β-gal-positive A549 cells declined after the cells were released from PF-573228 inhibition. **(B)** The proportion of SA-β-gal-positive A549 cells was ~80% when cells were exposed to PF-573228, and only 25% after PF-573228 withdrawal. **(C)** Initially, the cells were senescent, and proliferation ceased in the three lung cancer cell lines with PF-573228 treatment. After PF-573228 withdrawal, the A549 cells grew exponentially, H1299 cells grew linearly, and H460 cells continued to exhibit cessation of division. **(D)** When cells were exposed to PF-573228, cyclin B1 expression level was extremely low. After the cells were released from PF-573228 inhibition, cyclin B1 levels gradually increased in A549 cells and H1299 cells. However, cyclin B1 remained at low levels in H460 cells after PF-573228 withdrawal. ^*^P < 0.05.

In this study, FAK signaling downregulated the expression of p53, and inhibition of FAK signaling upregulated the expression of p53 in A549 cells (Figures 4C,D, 5A). The A549 cells that entered senescence due to PF-573228 administration exhibited regrowth and return to a non-senescent state after withdrawal of PF-573228 from the culture (Figures 6A–C). The proliferative activity of A549 cells was based on cyclin B1 expression after the cells were incubated in PF-573228-free medium (Figure 6D).

### Restoration of Lamin a and Lamin C Expressions in Senescent Lung Cancer Cells After PF-573228 Withdrawal

**Figure 7:**
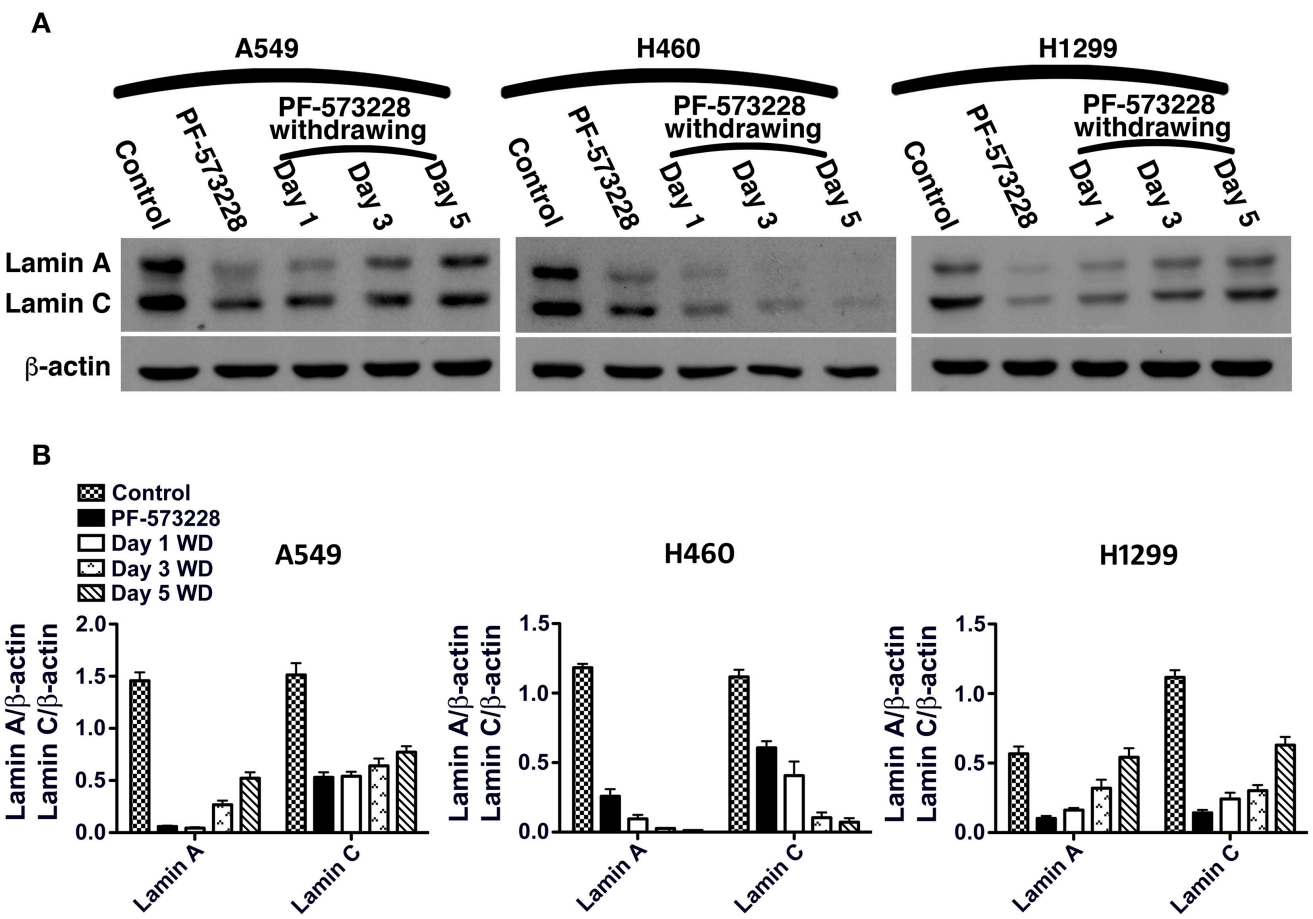
Restoration of lamin A and lamin C expressions in lung cancer cells after PF-573228 withdrawal (WD). **(A)** The senescent cells were released from PF-573228 inhibition for the indicated period. Western blot analysis revealed that the expression levels of lamin A and lamin C were gradually restored in A549 cells and H1299 cells. However, the lamin A and lamin C levels remained lower in H460 cells when senescent H460 cells were incubated in PF-573228-free medium. **(B)** The expression levels of lamin A and lamin C in A549 cells increased after the cells were released from PF-573228 inhibition. However, H460 cells expressed lower levels of lamin A and lamin C when exposed to PF-573228, and lamin A and lamin C expression in H460 cells remained lower when PF-573228-treated cells were cultured in PF-573228-free medium. Lamin A and lamin C levels in H1299 cells decreased when cells were exposed to PF-573228. After PF-573228 withdrawal, the expression levels of lamin A and lamin C gradually increased in H1299 cells previously treated with PF-573228.

**Figure 8:**
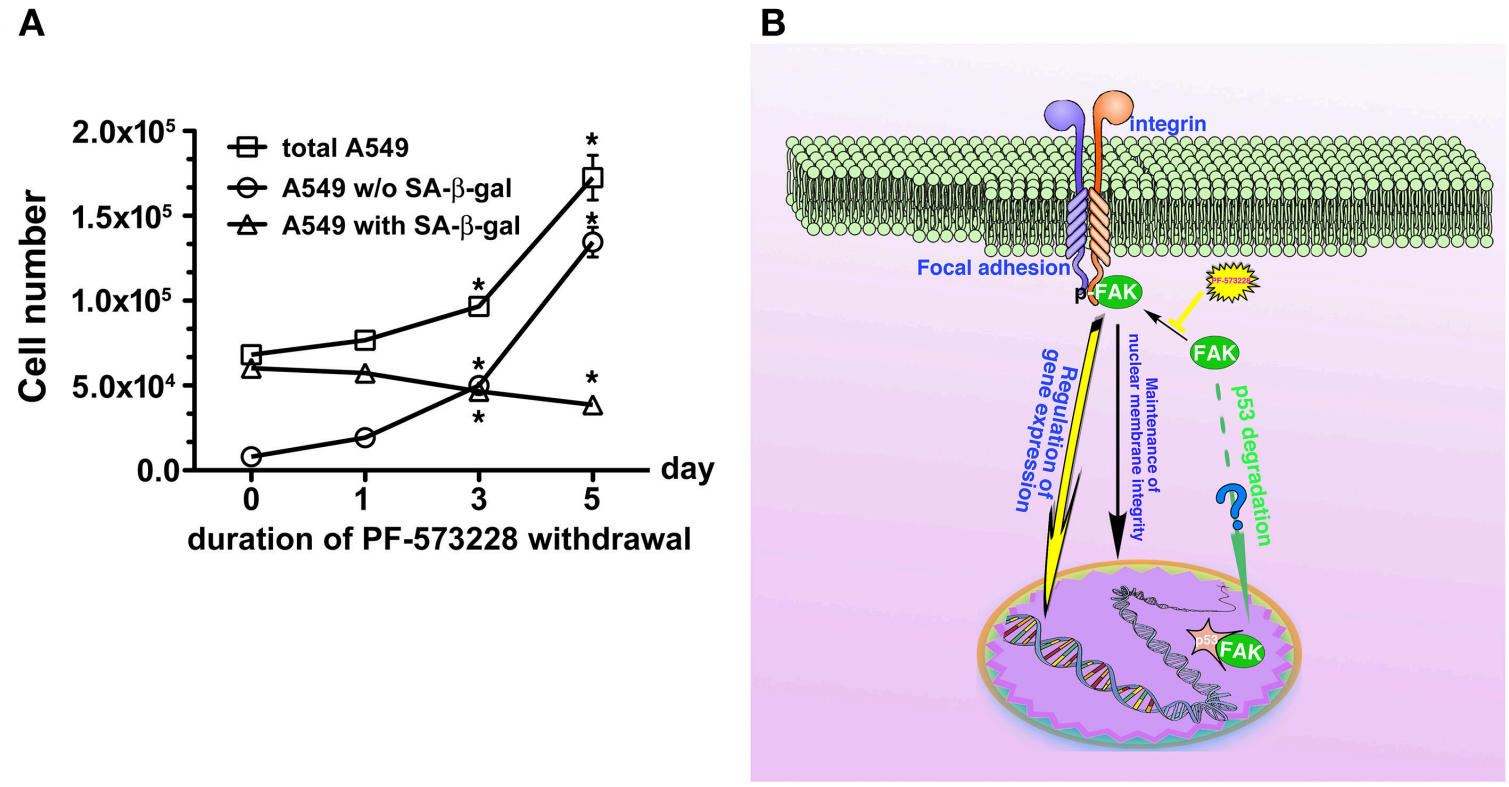
Disruption of FAK signaling with cellular senescence. Reactivation of FAK signaling was observed in A549 cells in which senescence was induced by 5 days of PF-573228 treatment after PF-573228 withdrawal. **(A)** The proportion of SA-β-gal-positive A549 cells declined to half when the senescent cells were cultured in PF-573228-free medium. In addition, A549 cells grew exponentially after A549 cells were released from inhibition by 10 μM PF-573228. ^*^P < 0.05. **(B)** The proposed scheme shows that integrin-based signaling activates FAK to trigger cell proliferation, to manage lamin A/C expression to maintain nuclear shape and program anti-senescence. Blockade of FAK signaling by PF-573228 induced cell cycle arrest and senescence.

We then tested whether senescence in lung cancer cells with FAK inhibition was reversible or irreversible. The three lung cancer cells lines were cultured in medium containing 10 μM PF-573228 to induce cell senescence in a 5-day induction course, after which the expressions of lamin A and lamin C decreased (Figure 7A). After the induction of senescence, PF-573228 withdrawal was scheduled over 6 days. Lamin A and lamin C expressions in A549 cells and H1299 cells gradually recovered after PF-573228 withdrawal (Figures 7A,B). By contrast, lamin A and lamin C levels in H460 cells remained lower when senescent H460 cells were incubated in PF-573228-free medium (Figures 7A,B). After PF-573228 withdrawal, senescent A549 cells escaped from senescence, as the SA-β-gal-positive A549 cell population declined to nearly half by the fifth day of PF-73228 withdrawal (Figure 8A).

## Discussion

FAK is a signaling mediator of integrin-based signaling and is associated with epidermal growth factor receptor (EGFR) signaling (23, 53). FAK-associated cross-talk between EGFR and integrin pathways have been shown to lead to tumor growth and metastasis in lung cancer (39, 53–55). Phosphorylation at tyrosine 576/577 (p-FAK) has been reported to result in catalytic activity and to be involved in tumor cell proliferation and metastasis (2, 12, 15, 16). Therefore, inhibition of the enzymatic function of FAK has been proposed to be a therapeutic strategy to limit tumor growth, angiogenesis, and metastasis (5, 7, 16, 23). In the present study, we tested the pharmacological effect of PF-573228 on inhibiting FAK activity and limiting lung cancer cell growth. When lung cancer cells were treated with PF-573228, an abnormal nuclear shape was observed. A similar cytological phenomenon has been reported in previous studies (56), however the molecular mechanism has not been clearly elucidated. In addition, nuclear lobulation and distorted nuclear morphology have been reported in cells with the LNMA mutation or lamin A/C downregulation (41–43). The LNMA mutation or lamin A/C downregulation has been shown to result in nuclear distortion with a pathogenic tendency to develop aging and senescence (8, 30, 42, 57). The nuclear deformity in PF-573228-treated lung cancer cells (Figures 2B, 3A–C) supports a pathophysiological impact from the inactivation of FAK signaling to downregulate lamin A/C.

In this study, we examined the expressions of lamin A/C and assayed SA-β-gal activity in lung cancer cells exposed to PF-573228. Our experimental results demonstrated that FAK inhibition and FAK depletion elicited similar downregulation of lamin A/C, upregulation of p53, and cellular senescence (Figures 4A,C,D, 5A,C). These results imply that FAK signaling regulates the expression of lamin A/C to maintain a regular nuclear shape and activate anti-senescence programs (Figure 8B). The finding that FAK signaling affects lamin A/C expressions and influences the cellular context in which lamin A/C organizes the nuclear architecture is an important biological theme. The degradation of lamin A/C has recently been reported to be regulated by Akt1 or cdk5 signaling (30, 58). Akt signaling was shown to slightly alter the amount of lamin A/C in cells, but this small change in lamin A/C expressions did not seem to have a notable effect on nuclear shape. On the other hand, nuclear FAK has also been shown to act as a transactivator to regulate gene expressions and stem cell differentiation rather than stem cell renewal (59). In addition, nuclear FAK and Oct-4 have been shown to coordinate gene expression programming with the expression of Oct-4 in stem cell renewal. However, the role of nuclear FAK in gene expression programming does not seem to be associated with changes in lamin A/C expressions. Furthermore, we found that PF-573228 treatment does not dramatically affect nuclear translocation of FAK in A549 cells (Figure S5). This implied that FAK-mediated signaling to maintain lamin A/C expression may not be through transcriptional regulation. By contrast, inactivation of FAK signaling or nestin silencing (30) has been shown to significantly downregulate lamin A/C and cause round or oval nuclei to become lobulated or irregular in shape. Our results indicated that FAK-mediated signaling is crucial to maintain nuclear shape and, potentially, for chromatin reorganization.

In addition to the downregulation of lamin A/C, this study showed that FAK inhibition-mediated p53 upregulation also played a crucial role in cellular senescence, and that p53 was increased during FAK inhibition either by a small compound or shRNA-mediated downregulation. Lim et al. demonstrated that nuclear FAK could promote p53 downregulation via enhanced Mdm2-dependent p53 ubiquitination in a kinase-independent manner (11). However, in our study, both the amount of FAK protein and its enzymatic function affected the expression level of p53. PF-573228 treatment suppressed the enzymatic activity of FAK but did not significantly affect its abundance. However, an obvious effect on cell senescence was observed in the inhibitor treatment group. This result implies an important role of FAK enzymatic function in suppressing senescence. Downstream signaling such as the PI3K/Akt axis may play a critical role in modulating Mdm2 function and p53 regulation, and p53 activation may suppress cell proliferation and further trigger senescence. However, FAK inhibition also repressed the proliferation of p53 null cancer cells such as H1299 cells and induced senescence. FAK inhibition also reduced lamin A/C expressions in H1299 cells, with changes in chromatin integrity followed by the induction of senescence. These observations indicate that the induction of cellular senescence by perturbations in lamin A/C-mediated chromatin alterations is independent of p53 (30, 34, 60).

Downregulation or degradation of lamin A/C and upregulation of p53 by PF-573228 treatment or FAK depletion are the main causes of cellular senescence. Baell et al. reported that inhibition of histone acetyltransferase could induce cellular senescence (26), and that the pharmacological effects of VM-8014 and VM-1119 on chromatin remodeling caused cellular senescence (26). The induction of senescence by PF-573228, VM-8014, and VM-1119 may also be due to defective chromatin remolding. Because FAK and lamin A/C are also involved in chromatin remodeling (43, 55, 61), this cellular senescence is likely to be reversible (Figures 6A–C, 8A). Therefore, when the enzymatic activity of FAK is restored, lamin A/C and cyclin B1 expression levels recover (Figures 7A,B, 6D).

Inhibition of FAK signaling may have a therapeutic role in limiting cancer cell growth. In the present study, we demonstrated that disruption of the FAK signaling pathway led to cellular senescence in lung cancer cells. We also tested the sensitivity of human normal lung epithelial cells, BEAS-2B, to PF-573228 treatment. It appeared that a high concentration of PF-573228 could attenuate the propagation of BEAS-2B cells. However, the BEAS-2B cells cultured in medium containing serum still underwent cell cycle progression with a low proliferative rate (Figure S6A). This implies that oncogene addiction occurs in lung cancer cells for FAK signaling (54). We also evaluated whether FAK inhibition causes cellular senescence in BEAS-2B cells. The results showed that a high dose of PF-573228 treatment promoted cellular senescence in BEAS-2B cells (Figures S6B,C). However, the ratio of SA-β-gal positive cells was < 3% (Figure S6C). This implies that normal cells are more insensitive to high concentrations of PF-573228 than lung cancer cells and FAK inhibitors have a therapeutic potential for cancer treatment. However, there was no evidence showing that FAK signaling can result in anti-senescence and convert senescent cells to non-senescent cells upon FAK inhibitor withdrawal in A549 cells (Figures 8A,B). We calculated the ratios of SA-β-gal-positive and SA-β-gal-negative A549 cells after PF-573228 withdrawal and plotted curves with the timing of PF-573228 withdrawal. The slope of the curve for SA-β-gal-negative cells over 5 days indicated a reduction of 5,402 cells per day in the linear variation of SA-β-gal-negative cell numbers (Figure 8A). SA-β-gal-negative cells increased exponentially after PF-573228 withdrawal in A549 cells, and the curves of the SA-β-gal-negative cell growth were convergent with the growth curve of total A549 cells (Figure 8A). These results may be due to reversion of some of the senescent cells to non-senescent cells, as described in the schematic representation of FAK signaling in anti-senescence and PF-573228 treatment signaling cellular senescence (Figure 8B).

Previously, therapeutic outcomes were measured in terms of anti-angiogenesis, anti-proliferation, and anti-invasion (16, 39, 62). In this study, FAK inhibition limited lung cancer cell propagation by inducing cellular senescence (Figure 8B). Driving cell senescence programing is a new trend for the treatment of tumor diseases (26), as this therapeutic approach does not chemically elicit genomic evolution in cancer cells and does not severely damage non-cancer cells (63). Although cellular senescence does not kill tumor cells, limiting cancer growth could eliminate cancer cell malignancy. However, cellular senescence is an inducer of autophagy (64) and increases susceptibility to cell-mediated cytotoxicity by activated killer cells (65). Furthermore, FAK inhibition also increases immune surveillance (66). Consequently, FAK appears to be an attractive target for pharmacological strategies for cancer therapy. Our data reveal a signaling pathway for senescence and support a therapeutic strategy for cancer.

## Author Contributions

H-HC, Y-YZ, and C-JY conceptualized and designed this study. P-HW devised the methodology. H-HC, P-HW, and Y-YZ performed the experiments. S-WN and Y-YZ performed formal analysis. M-SH and MH provided the resources. H-HC and Y-YZ wrote the draft. M-SH, MH, and C-JY reviewed and edited the manuscript. All authors reviewed the manuscript.

### Conflict of Interest Statement

The authors declare that the research was conducted in the absence of any commercial or financial relationships that could be construed as a potential conflict of interest.
