# Supplementary material for: Inhibition of FAK Signaling Elicits Lamin A/C-Associated Nuclear Deformity and Cellular Senescence
Source: Front Oncol. 2019 Jan 30;9:22. doi: 10.3389/fonc.2019.00022 (PMC6363943; doi:10.3389/fonc.2019.00022)
Supplement: Supplementary file 1 [file Data_Sheet_1.docx]

Supplementary Material

Inhibition of FAK Signaling Elicits Lamin A/C-associated Nuclear Deformity and Cellular Senescence

Hsiang-Hao Chuang, Pei-Hui Wang, Sheng-Wen Niu, Yen-Yi Zhen, Ming-Shyan Huang, Michael Hsiao and Chih-Jen Yang*

*** Correspondence:** Chih-Jen Yang: [chjeya@cc.kmu.edu.tw](mailto:chjeya@cc.kmu.edu.tw)

1. **Supplementary Figures and Table**

**1.1 Supplementary Table**

**Supplementary Table S1: Used materials including provider source, identifier and working concentrations.**

| **RAGENT or RESOURCE** | **SOURCE** | **IDENTIFIER** | **WORKING STATUS** |
| --- | --- | --- | --- |
| **Antibodies** |  |  |  |
| **Anti-beta-Actin** | **Sigma-Aldrich** | **Cat# SI-A5441-.2 ml** | **5k dilution** |
| **Anti-cyclin B1** | **Cell Signaling Technology** | **Cat# 4138** | **1k dilution** |
| **Anti-emerin (FL-254)** | **Santa Cruz** | **Cat# sc-15378** | **500 dilution** |
| **Anti-FAK (D-1)** | **Santa Cruz** | **Cat# sc-271126** | **500 dilution** |
| **Anti-lamin A/C** | **GeneTex** | **Cat# GTX101127** | **1k dilution** |
| **Anti-p53** | **Cell Signaling Technology** | **Cat# 9282** | **1k dilution** |
| **Anti-paxillin (A-5)** | **Santa Cruz** | **Cat# sc-390738** | **500 dilution** |
| **Anti-phospho-paxillin(Tyr31)-R** | **Santa Cruz** | **Cat# sc-14035-R** | **500 dilution** |
| **Anti-Phospho-FAK (Tyr397)** | **Cell Signaling Technology** | **Cat# 3823** | **500 dilution** |
| **Anti-Phospho-FAK (Tyr576/577)** | **Cell Signaling Technology** | **Cat# 3281** | **500 dilution** |
| **Donkey anti-Mouse IgG (H+L) Secondary Antibody, Alexa Fluor 488** | **Invitrogen** | **Cat# R37114** |  |
| **Goat anti-Rabbit IgG (H+L) Cross-Adsorbed Secondary Antibody, Alexa Fluor 488** | **Invitrogen** | **Cat# A-11008** |  |
| **Cell lines** |  |  |  |
| **A549** | **ATCC** | **ATCC® CCL-185** | **RPMI 1640 + 10%FBS** |
| **BEAS-2B** | **ATCC** | **ATCC® CRL-9609** | **RPMI 1640 + 10%FBS** |
| **H1299** | **ATCC** | **ATCC® CRL-5803** | **RPMI 1640 + 10%FBS** |
| **H460** | **ATCC** | **ATCC® HTB-177** | **RPMI 1640 + 10%FBS** |
| **HEK293** | **ATCC** | **ATCC® CRL-1573** | **DMEM + 10%FBS** |
| **Chemicals, Enzymes and Materials** |  |  |  |
| **4',6-diamidino-2-phenylindole (DAPI)** | **Sigma-Aldrich** | **Cat# D9542-1MG** | **0.2 μg/mL** |
| **BEGM BulletKit Growth Media** | **Lonza** | **Cat# CC-3170** |  |
| **Dulbecco's Modified Eagle Medium** | **Gibco** | **Cat# 12100-061** |  |
| **Fetal bovine serum** | **Gibco** | **Cat# 10437-028** |  |
| **Lipofectamine 2000** | **Invitrogen** | **Cat# 11668019** |  |
| **PF-573228** | **TOCRIS** | **Cat# 3239** |  |
| **Phalloidin-TRITC** | **Sigma-Aldrich** | **Cat# P1951** | **1k dilution** |
| **Puromycin** | **InvivoGen** | **Cat# ant-pr-1** | **2μg/mL** |
| **RIPA Lysis Buffer, 10X** | **Millipore** | **Cat# 20-188** |  |
| **RPMI medium1640** | **Gibco** | **Cat# 31800-089** |  |
| **PhosSTOP™** | **Roche** | **ROC-04906845001** |  |
| **SIGMAFAST™ Protease Inhibitor Cocktail Tablets** | **Sigma-Aldrich** | **SI-S8830-20TAB** |  |
| **Western Lightning Plus-ECL** | **Perkin Elmer** | **Cat# 50-904-9326** |  |
| **X-Gal Stock Solution** | **Millipore** | **Cat# BG-3-G** |  |
| **Recmbinant DNA** |  |  |  |
| **Luciferase shRNA** | **RNAi Core Facility of Academia Sinica** |  |  |
| **pCMVdeltaR8.91** | **RNAi Core Facility of Academia Sinica** |  |  |
| **pMD.G** | **RNAi Core Facility of Academia Sinica** |  |  |
| **shFAK #1** | **RNAi Core Facility of Academia Sinica** | **TRCN 000121207** |  |
| **shFAK #2** | **RNAi Core Facility of Academia Sinica** | **TRCN 000121308** |  |
| **Other** |  |  |  |
| **Leica DMI6000 B microscope** | **Leica** | **N/A** |  |
| **Olympus FV1000 confocal laser scanning microscope** | **Olympus** | **N/A** |  |

**1.2 Supplementary Figures**


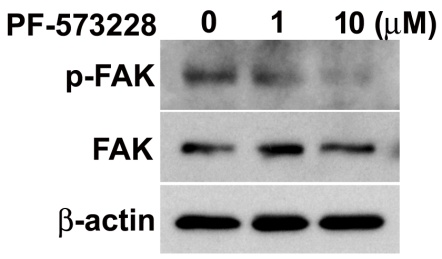


**Supplementary Figure S1:** **PF-573228 treatment reduces FAK Tyr397 phosphorylation.** FAK expression levels and the status of phosphorylation of FAK at tyrosine 397, were quantified by Western blot analysis after treatment of A549 cells with PF-573228 for 3 days.


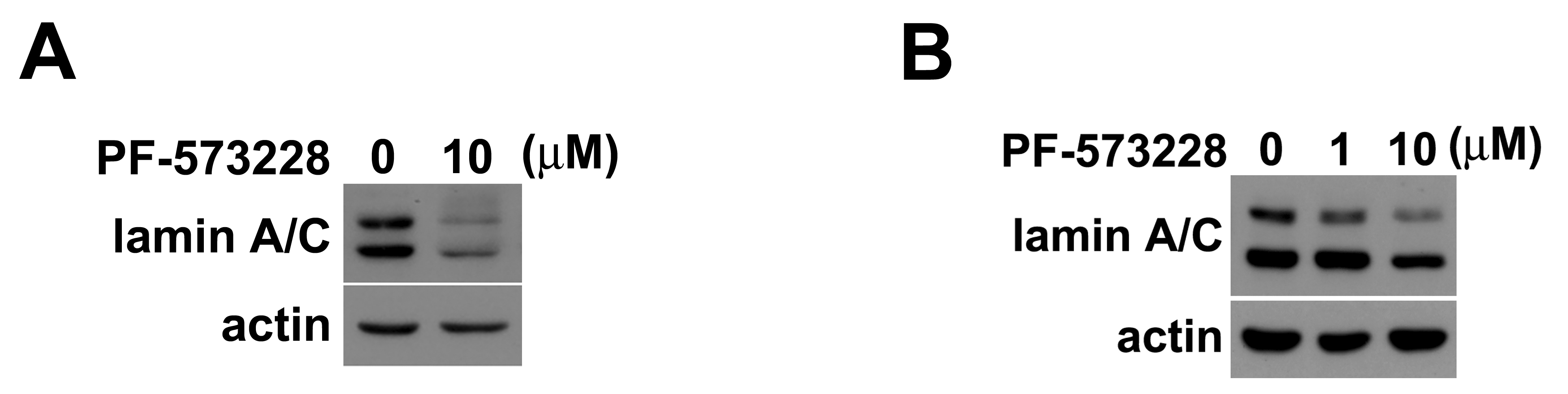


**Supplementary Figure S2:** **PF-573228 treatment downregulates lamin A/C expression in lung cancer cells.** (A) H460 and (B) H1299 cells were treated with the indicated concentrations of PF-573228 for 96 h and harvested for Western blotting with individual antibodies against lamin A/C and β-actin. H460 and H1299 cells with 10 μM PF-573228 treatment had decreased amounts of lamin A and lamin C.


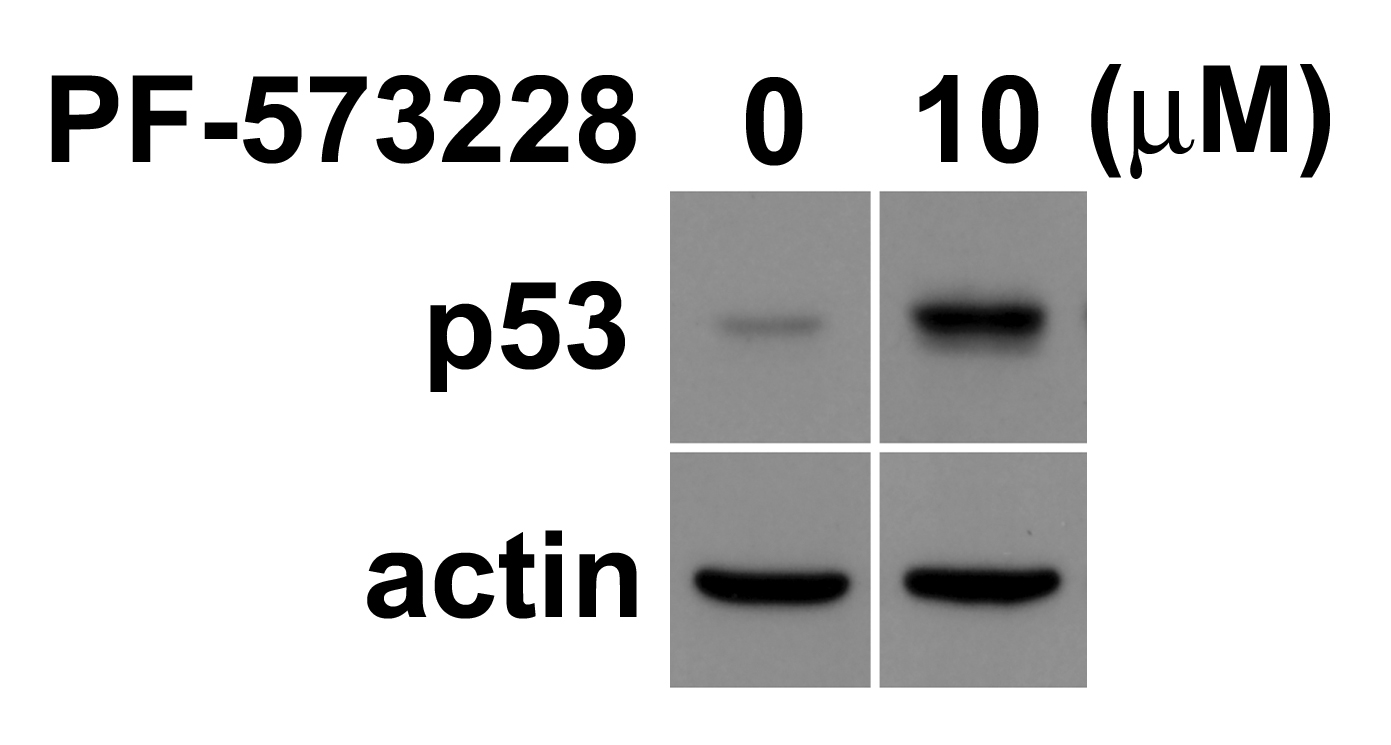


**Supplementary Figure S3: PF-573228 treatment upregulates p53 expression in H460 cells.** H460 cells were treated with or without 10 μM PF-573228 for 96 h and harvested for Western blotting with antibodies against p53 and β-actin. p53 expression levels were induced in the H460 cells exposed to 10 μM PF-573228.


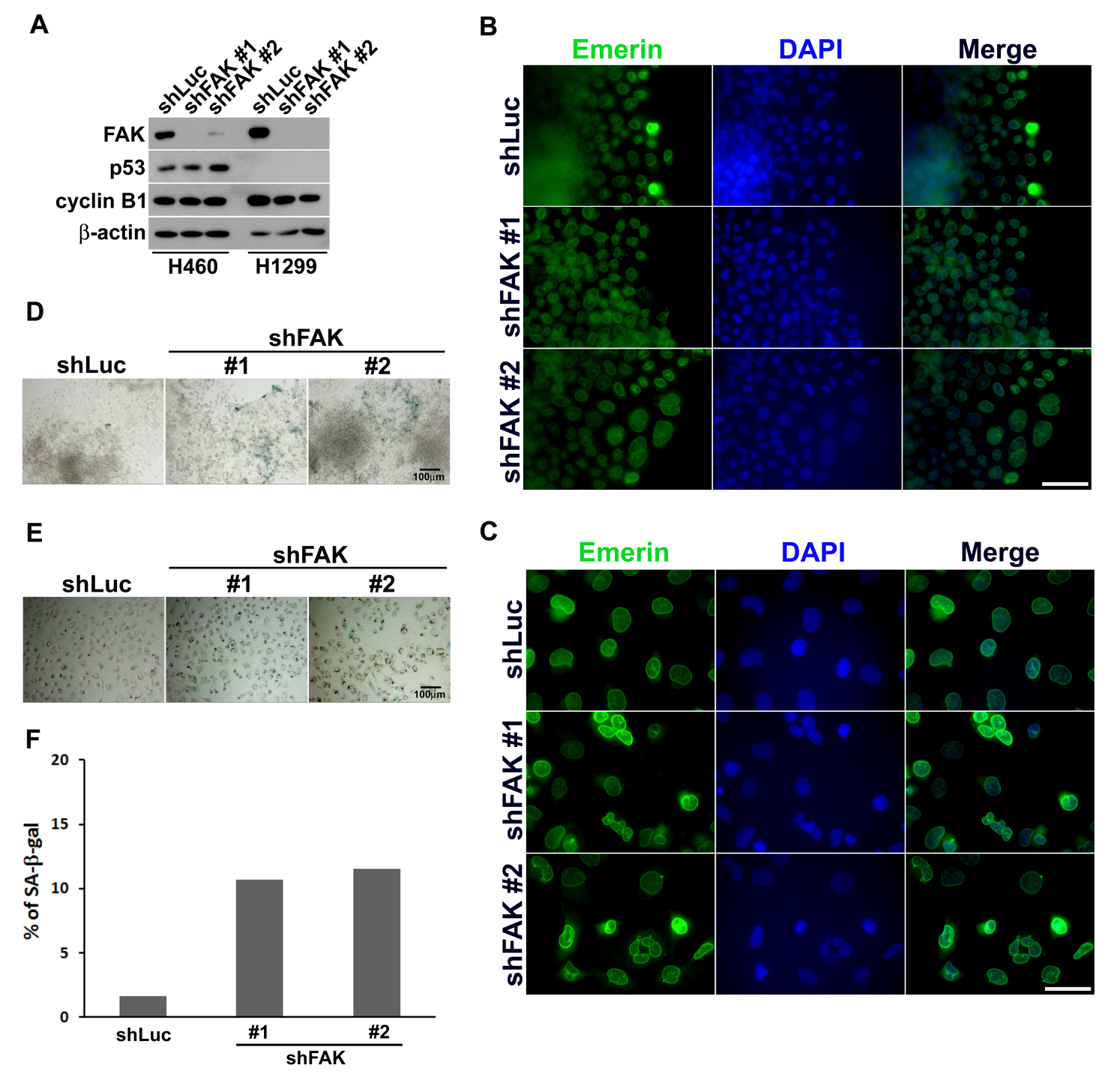


**Supplementary Figure S4: FAK depletion elicits nuclear deformity and cellular senescence in lung cancer cells.** (A) H460 and H1299 cells with FAK depletion by shRNA were harvested for Western blotting with the indicated antibodies. FAK and cyclin B1 levels were reduced, and p53 expression level was increased. (B) H460 and (C) H1299 cells with FAK depletion by shRNA were seeded and incubated for 7 days. The cells were fixed and stained with an antibody against emerin (green) to visualize the nuclear shape. Cells with FAK depletion were slightly larger, with a higher proportion of deformed nuclei, whereas mostly oval shaped nuclei were present in cells without FAK depletion. (Scale bar, 50 μm) (D) 1.5 x 10^3^ H460 and (E) H1299 cells with FAK depletion by shRNA were seeded and incubated for 7 days followed by senescence associated β-galactosidase (SA-β-gal) staining to detect senescent cells. SA-β-gal positive cells were sporadically visible in H460 and H1299 cells with shLuc. In contrast, more SA-β-gal positive cells were observed among cells with shFAK. (F) The ratios of SA-β-gal-positive H1299 cells were measured. More than 1000 cells were counted for each group.


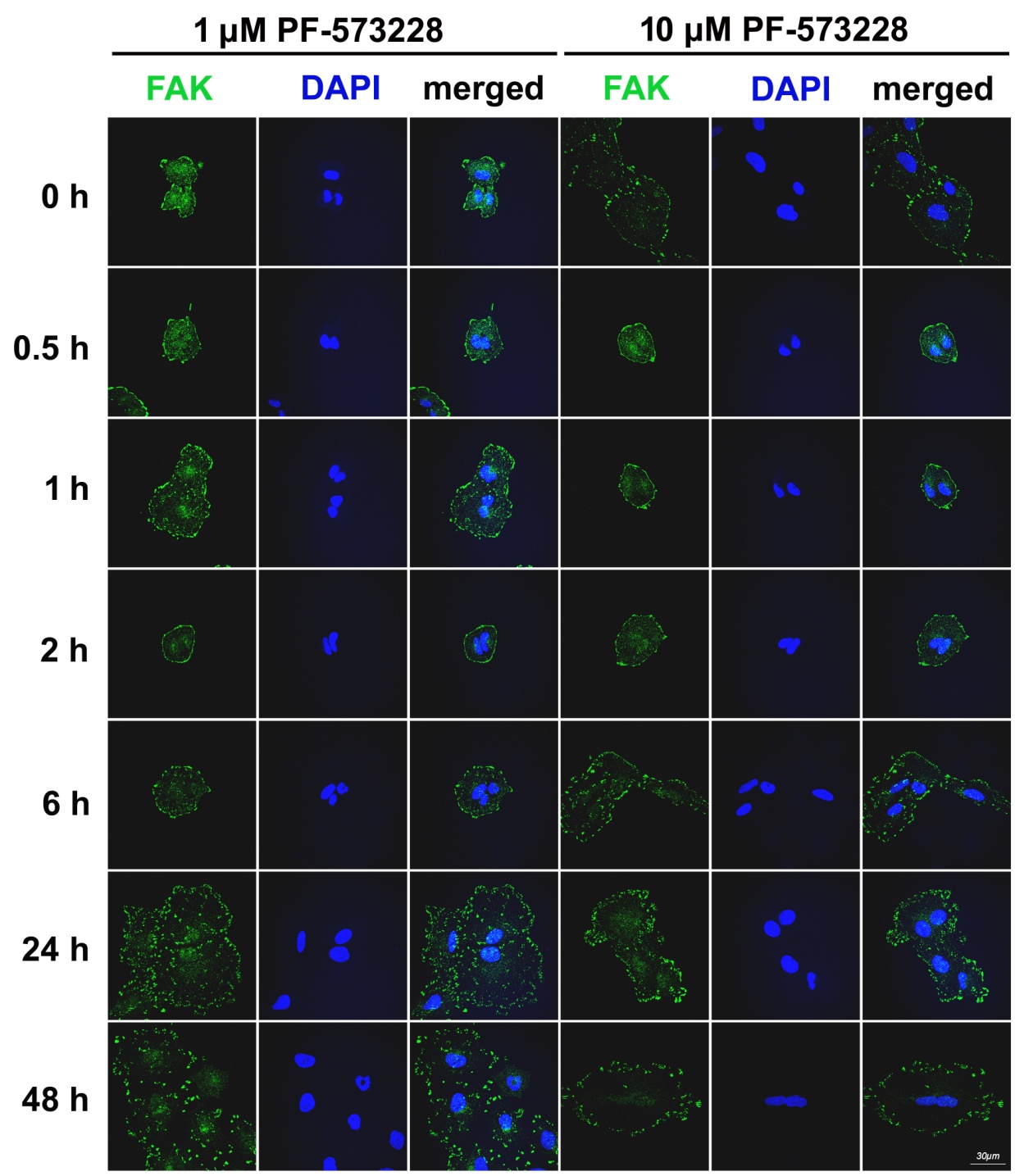


**Supplementary Figure S5: PF-573228 treatment does not dramatically change the cellular localization of FAK.** A549 cells were treated with 1μM or 10 μM PF-573228 for the indicated periods and followed by fixation. The fixed cells were stained with an antibody against FAK to visualize the FAK distribution (green) using confocal laser scanning microscope. Nuclei were visualized with DAPI staining (Scale bar, 30 μm).


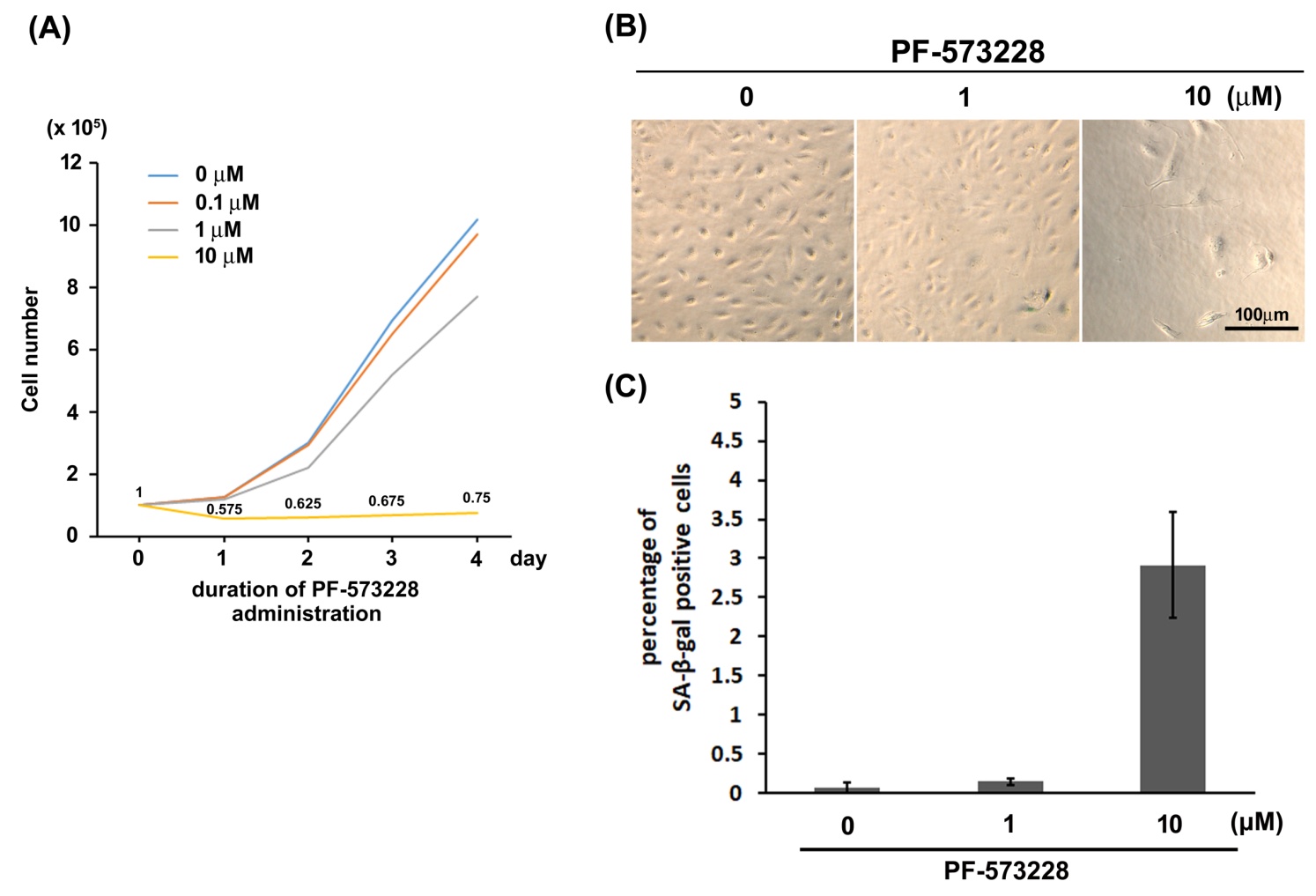


**Supplementary Figure S6: PF-573228 treatment attenuates the cell growth and has little effect on cellular senescence in BEAS-2B cells.** (A) BEAS-2B cells were cultured in medium containing various concentrations of PF-573228 for indicated periods of time. The cell numbers of the treated cells were counted and plotted in the bar chart. Administration of PF-573228 at 10 μM to BEAS-2B effectively suppressed cell growth *in vitro*. However, BEAS-2B cells treated with PF-573228 still underwent cell cycle progression with a very low proliferation rate. (B) 1.5 x 10^3^ BEAS-2B cells were treated with indicated concentration of PF-573228 for 7 days followed by senescence associated β-galactosidase (SA-β-gal) staining to detect senescent cells. (Scale bar, 100 μm) (C) The ratios of SA-β-gal-positive BEAS-2B cells were measured. Data were presented as mean ± SEM of three independent experiments.
